# Significance of Tumor Mutation Burden Combined With Immune Infiltrates in the Progression and Prognosis of Advanced Gastric Cancer

**DOI:** 10.3389/fgene.2021.642608

**Published:** 2021-07-09

**Authors:** Xiong Guo, Xiaolong Liang, Yujun Wang, Anqi Cheng, Han Zhang, Chuan Qin, Ziwei Wang

**Affiliations:** ^1^Department of Gastrointestinal Surgery, The First Affiliated Hospital of Chongqing Medical University, Chongqing, China; ^2^Department of Pathology, Daping Hospital, Army Military Medical University, Chongqing, China; ^3^Department of Digestive Oncology, Three Gorges Hospital, Chongqing University, Chongqing, China; ^4^Department of Gastrointestinal Surgery, Three Gorges Hospital, Chongqing University, Chongqing, China

**Keywords:** advanced gastric cancer, tumor mutation burden, immune infiltration, prognosis, bioinformatics analysis

## Abstract

Gastric cancer (GC) is a serious malignant tumor with high mortality and poor prognosis. The prognosis and survival are much worse for advanced gastric cancer (AGC). Recently, immunotherapy has been widely promoted for AGC patients, and studies have shown that tumor mutation burden (TMB) is closely related to immunotherapy response. Here, RNA-seq data, matched clinical information, and MAF files were downloaded from the cancer genome atlas (TCGA)-STAD project in the TCGA database. The collation and visual analysis of mutation data were implemented by the “maftools” package in R. We calculated the TMB values for AGC patients and divided the patients into high- and low-TMB groups according to the median value of TMB. Then, the correlation between high or low TMB and clinicopathological parameters was calculated. Next, we examined the differences in gene expression patterns between the two groups by using the “limma” R package and identified the immune-related genes among the DEGs. Through univariate Cox regression analysis, 15 genes related to prognosis were obtained. Furthermore, the two hub genes (APOD and SLC22A17) were used to construct a risk model to evaluate the prognosis of AGC patients. ROC and survival curves and GEO data were used as a validation set to verify the reliability of this risk model. In addition, the correlation between TMB and tumor-infiltrating immune cells was examined. In conclusion, our results suggest that AGC patients with high TMB have a better prognosis. By testing the patient’s TMB, we could better guide immunotherapy and understand patient response to immunotherapy.

## Introduction

Gastric cancer (GC) is a common malignant tumor worldwide, with the fifth and third highest morbidity and mortality, respectively, of all cancers (Chen, 2016). This disease seriously threatens human health. The 5-year survival rate of advanced gastric cancer (AGC) is less than 25% (Ajani et al., 2017). In recent years, with the improvement of diagnosis and treatments, there has been a steady decline in the incidence and mortality rates of this cancer. However, despite the decline in incidence in most countries, clinicians are still expected to see more cases of GC in the future due to the aging population. On the other hand, because the onset of gastric cancer is insidious, it is frequently at an advanced stage at diagnosis, and resulting in a high mortality rate (Cascinu, 2020). At present, the best treatment for patients with GC is surgery, but aging patients cannot tolerate surgery, and in some cases the tumor is discovered too late for surgery to be effective. Therefore, palliative care is particularly important for these patients. In addition to radiotherapy and chemotherapy, immunotherapy has made great progress in recent years, and bringing hope to patients with AGC.

Traditionally, patients with advanced inoperable gastric cancer are treated with sequential chemotherapy, mainly platinum and fluoropyrimidine combination drugs (Song et al., 2017). However, the median survival is still less than 1 year. Recently, immune checkpoint inhibitors (ICIs), such as anti-programmed cell death-1 (PD-1) or programmed cell death ligand-1 (PD-L1) monoclonal antibodies, have improved the overall survival (OS) of various types of cancers, including AGC (Kim and Oh, 2018). To date, two anti-PD-1 inhibitors have been approved for AGC in Japan: nivolumab as third- or later-line treatment for AGC and pembrolizumab for previously treated patients with microsatellite instability-high tumors (Kawazoe et al., 2020). However, some gastric cancers may not be sensitive to immune checkpoint inhibitor monotherapies, so patients with gastric cancer may require combination therapy to improve the response to anti-PD-1 therapy. Therefore, methods to predict and improve patient response to immunotherapy or novel treatment methods are highly desired for AGC (Cascinu, 2020). A recent study suggested that predicting the response to immunotherapy on the basis of the tumor mutation burden (TMB) load may be a new opportunity (Morrison et al., 2018).

Tumor mutation burden is defined as the total number of somatic gene coding errors, base insertions, substitutions, or deletion errors detected per million bases (Yarchoan et al., 2017). Mutations in driver genes can lead to cancers. However, if a large number of somatic cell mutations occur, new antigens will be produced to activate CD8+ cytotoxic T cells, and triggering T-cell-mediated antitumor activity (Bi et al., 2020). Therefore, as the TMB increases, more new antigens are produced, and the tumors are more easily recognized by immune cells in the tumor microenvironment. TMB was used as a biomarker for anti-PD-1 treatment in colorectal cancer, and a higher TMB was associated with a better response to immunotherapy (Le et al., 2015). Recently, Tian et al. (2020) constructed a novel TMB estimation model that can be used as a prognostic biomarker for patients with non-small cell lung cancer. TMB can predict not only the response to immunotherapy but also patient survival. However, there are few studies on the relationship between TMB and immune infiltration in AGC.

In this study, we calculated the TMB of 338 AGC patients with complete clinical information, revealing the mutation characteristics of AGC patients. Then, we studied the correlation between the clinicopathological parameters and the normalized TMB value. Two TMB-related gene signatures were used to construct a risk model that could predict the survival of AGC patients. Moreover, we explored the relationship between TMB and the tumor microenvironment and provided new targets for immunotherapy for GC.

## Materials and Methods

### Data Acquisition and Processing

The transcriptome data were obtained using the Illumina (San Diego, CA, United States) HiSeq 2000 RNA sequencing platform, and the genetic mutation data were downloaded from the cancer genome atlas (TCGA) database^1^. The transcriptome profiles are HTseq-Count files. The mutation data are in Annotated Somatic Mutation format, and the workflow type is “VarScan2 Annotation.” Clinical data for the corresponding GC patients were also retrieved from the STAD project in TCGA database, which included age, tumor stage, sex, and survival information. The patient’s clinical information was provided in Supplementary Table 1. We excluded patients with incomplete clinical information and a survival time of less than 30 days and then selected patients with AGC for analysis based on the clinical information. The “maftools” package in R software was used to visually analyze the mutation annotation format (MAF) file (Mayakonda et al., 2018). Gene chip data of gastric cancer was downloaded from the NCBI (National Center for Biotechnology Information) GEO database as the data for the validation set. The chip number is GSE84437, submitted by Yong-Min Huh and others. The study included transcriptome results and complete clinical information of 433 gastric cancers. In addition, the list of immune-related genes was obtained from the resources section of the ImmPort database^2^.

### Calculation of the Tumor Mutation Burden

Tumor mutation burden was defined as the number of somatic coding insertion/deletion mutations and non-synonymous base replacements per megabase of the genome, and it was estimated by estimating the number of somatic mutations and dividing the total length of the exons. First, we used Perl scripts to extract tumor mutation data from AGC patient sequences and then used R software to calculate the TMB value according to the following formula for each patient:

T⁢M⁢B=S⁢n×1000000/n

where, Sn represents the absolute number of somatic mutations and n represents the number of exon bases with coverage depth ≥ 100× (Jiang et al., 2019). The calculated TMB value of the patient is provided in Supplementary Table 2.

### Prognostic Analysis of TMB Value

After calculating the TMB value for each patient, the TMB value was combined with the patient clinical information, including survival status and survival time. Then, all patients were assigned to either the high- or low-TMB group, with the median value of TMB as the cutoff. Kaplan-Meier (K-M) survival analysis and log-rank tests were performed to evaluate the difference in the OS rate between the above two groups. Additionally, we explored the relationship between TMB and clinical features, including sex, age, tumor grade, and TNM stage. The patients were divided into two groups according to clinical characteristics, and the Wilcoxon rank-sum test was used for statistical analysis.

### Identification of TMB-Related Differentially Expressed Genes and Functional Enrichment Analysis

The gene expression data from AGC patients were standardized by the “limma” R package, and then the DEGs between the high- and low-TMB groups were identified using the Wilcoxon test. | Log_2__–_fold change (FC)| > 1.0 and false discovery rate (FDR) < 0.05 were used as cutoffs to identify qualified DEGs for subsequent analyses, and volcano maps and heat maps were used for visual analysis using the “pheatmap” R package. In addition, we carried out gene ontology (GO) and kyoto encyclopedia of genes and genomes (KEGG) pathway functional enrichment analyses by using the “clusterProfiler” R package and visualized the enrichment results (Yu et al., 2012).

### Construction and Verification of Risk Score Model

We took the intersection of the previously obtained immune-related gene list with the TMB-related differential genes and obtained the immune genes that were differentially expressed in the low- and high-TMB groups. Since these genes are related to immunity and TMB in AGC, they were used for further analysis. First, univariate Cox regression analysis was used to identify candidate genes associated with survival. Next, the “glmnet” package in R software was used to further filter the risk model with least absolute shrinkage and selection operator (LASSO) Cox regression analysis. Finally, multiple Cox regression analysis was used to further screen the optimal prognostic genes for the construction of risk models, and a time-dependent receiver operating characteristic (ROC) curve was used to assess the accuracy of the constructed model (Guo et al., 2020). The expression of genes and the regression coefficients obtained in the regression model were used to calculate the patients’ risk scores. The calculation formula is as follows. Risk score (patients) = Σ Coefficient (gene *i*) * expression value (gene *i*). Where, n, i, coefficient, and expression value represent the number of selected genes, gene number, regression coefficient value, and gene expression value, respectively.

Meanwhile, the log-rank test was used to analyze the survival data between the low- and high-TMB groups. In addition, GSE84437 data were downloaded from the GEO database as a validation set, and the risk model was used to analyze the prognosis of gastric cancer patients. The clinical information of patients in the GSE84437 database was provided in Supplementary Table 3. A nomogram was constructed by gene expression based on this model to predict the different annual survival rates of patients for TCGA and GEO data.

### Evaluation of Immune Cell Infiltration

CIBERSORT is a deconvolution algorithm that combines the labeled genomes of different immune cell subpopulations to calculate the proportions of 22 immune cells in tissues. The 22 types of immune cells include various myeloid cells, NK cells, 3 types of B cells, and 7 types of T cells (Bi et al., 2020). In this study, we analyzed tumor immune cell infiltration in the tumor microenvironment of AGC patients in the low- and high-TMB groups. Samples with a CIBERSORT output *p*-value < 0.05 were screened for further analysis.

Furthermore, the tumor immune estimation resource (TIMER) web server was used to precalculate the abundance of six tumor-infiltrating immune subsets (Kang et al., 2020). The modules in TIMER were used to explore the association of immune infiltration with gene expression and survival outcomes in the current study^3^.

### Evaluation of the Value of Genes in the Model in a Pan-Cancer Panel

The cancer genome atlas pancancer data (ACC, BLCA, RCA, CESC, CCA, COAD, DLBC, GBM, HNSC, KIRC, KICH, KIRP, LGG, LAML, LIHC, LUSC, LUAD, MESO, OV, PAAD, PRAD, PCPG, READ, SKCM, SARC, TGCT, THYM, THCA, UCS, UCEC, and UVM), including RNA-Seq, stemness scores based on mRNA (RNAss) and DNA methylation (DNAss) and matched clinical information, were downloaded from the Xena browser^4^. We calculated the expression of APOD and SLC22A17 in the 33 cancers in the pancancer dataset, and through univariate Cox regression analysis, the risk values of these two genes for these 33 cancers were calculated. The Pearson correlation test method was used to calculate the correlation between gene expression and stromal scores, RNAss, and DNAss of 33 different cancer types based on the ESTIMATE algorithm. The drug responses to 262 FDA-approved drugs or drugs in clinical trials were included in the correlation analysis. The data were downloaded from the NCI-60 database, which contains data on 60 different cancer cell lines from 9 different tumors^5^ (Zhang X. et al., 2020).

### Statistical Analyses

All data were processed with Perl (**??**) and R (version 3.6.2) software. Survival analyses were performed using the K-M method and the log-rank test. Pearson’s correlation test was used for the correlation analysis between two groups. The Wilcoxon rank-sum test was used for differential analyses of subgroups. All statistical tests were two-sided, and *P* < 0.05 was considered statistically significant.

## Results

### Somatic Mutation Analysis in Advanced Gastric Cancer

**FIGURE 1:**
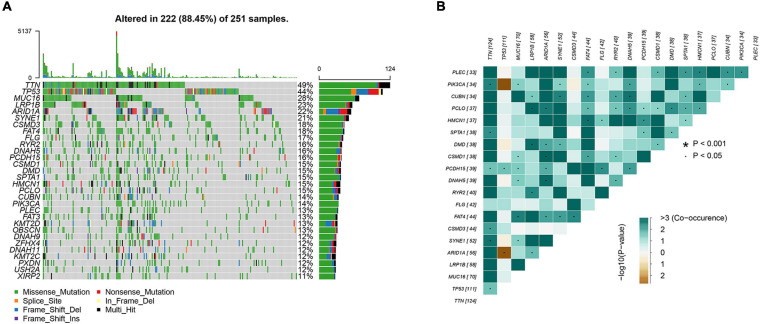
Analyses of somatic mutation profiles in advanced gastric cancer. **(A)** Waterfall plot of detailed mutation information of top 30 genes in each sample, with various color annotations to distinguish different mutation types. **(B)** Correlation between the top 20 mutated genes.

To identify somatic mutations in AGC patients in the TCGA database, we used the “maftools” package in R software to visually analyze the mutation data. Complete somatic mutation data were available for 251 AGC patients, of which 222 (88.45%) had somatic mutations. The 30 genes with the highest mutation rates in patients with AGC are displayed in the waterfall plot (Figure 1A) and include well-known cancer-related genes such as TTN (49%), TP53 (44%), and MUC16 (28%). Among them, missense mutations were the most common variant classification, single-nucleotide polymorphisms (SNPs) were the most common variant type, and *C* > T mutations accounted for the vast majority of single nucleotide variations (SNVs) (Supplementary Figures 1A–C). The maximum number of mutations in one sample was 5137 (Supplementary Figure 1D), and the median number of mutations was 90 (Supplementary Figure 1E). In addition, we showed the number of each variant in the different samples through box plots (Supplementary Figure 1F). And the correlation calculations for top 20 mutated genes are shown in Figure 1B. Moreover, we classified these mutant genes and identified their enrichment in different pathways (Supplementary Figure 1G) and mutations in all samples of AGC (Supplementary Figure 1H). The most mutated pathways were RTK-RAS (77/85, 90.59%), WNT (66/68, 97.06%), and NOTCH (57/71, 80.28%). In addition, 55.78% of the patients had mutations in the RTK-RAS pathway (140/251), 43.82% (110/251) had mutations in the WNT pathway, and 42.63% (107/251) had mutations in the NOTCH pathway. These are the key signaling pathways in cancer progression. The mutant genes in RTK-RAS, WNT, and NOTCH pathway in patients with AGC are shown in the waterfall chart, respectively (Supplementary Figures 1I–K).

### Correlation Between TMB and Clinicopathological Characteristics of AGC Patients

**FIGURE 2:**
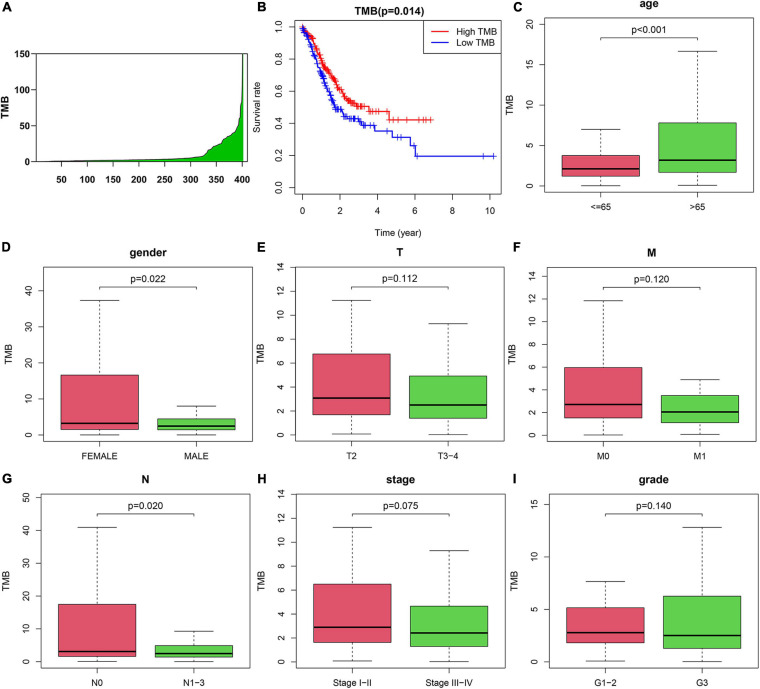
Correlation between tumor mutation burden (TMB) and clinicopathological characteristics of AGC patients. **(A)** TMB value in advanced gastric cancer samples. **(B)** Survival analysis between high-TMB and low-TMB patients. **(C–I)** Correlation between TMB and **(C)** age, **(D)** gender, **(E)** T, **(F)** M, **(G)** N, **(H)** Stage, and **(I)** grade of patients.

To explore the prognostic function of TMB, we calculated and visualized the TMB value of gastric cancer samples in the TCGA database (Figure 2A). Then, we divided patients into low-TMB and high-TMB groups according to the median value of TMB. The TMB values for each patient were shown in Supplementary Table 2. The survival rate of the two groups was plotted by using K-M curves. Interestingly, we found that the survival rate of patients in the high TMB group was superior to that of patients in the low TMB group (Figure 2B). To further investigate the correlation between TMB and the clinical characteristics of gastric cancer patients. We downloaded the clinical information and detected the relationship between TMB and clinical features. The results showed that TMB is positively correlated with patient age. In addition, TMB was negatively correlated with sex and N stage. It means female patients with age < 65 have less TMB value than the other people. In addition, patients with no lymph node metastasis might have less TMB. There were no correlations between TMB and T stage, M stage, stage, or tumor grade (Figures 2C–I).

### Variation in the Genes Related to TMB and Functional Analysis

**FIGURE 3:**
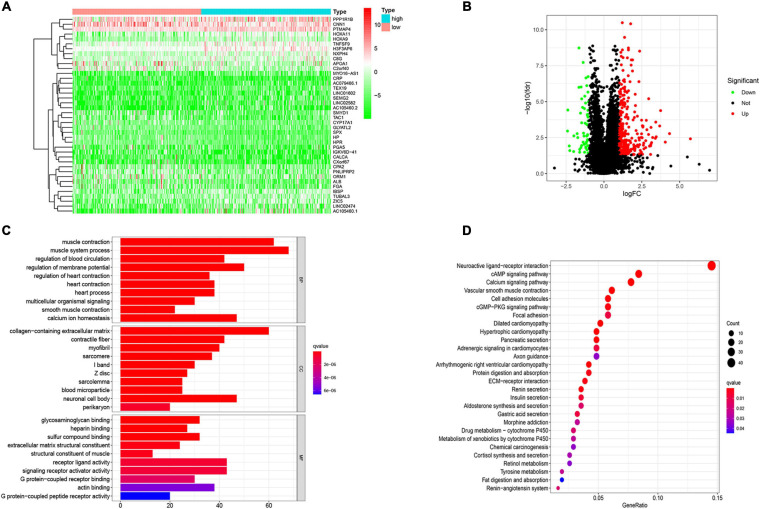
Variation in the genes related to TMB and functional analysis. **(A)** The differentially expressed genes (DEGs) related to TMB. **(B)** Volcano ma2p of DEGs. **(C)** Go and **(D)** KEGG analysis of DEGs related to TMB. The abscissa represents the number and proportion of genes, respectively.

One of the ways in which TMB functions is to affect gene expression. To obtain the DEGs related to TMB, we divided patients into a high TMB group and a low TMB group according to the median TMB value. Then, the “limma” package in R software was used to identify genes that were differentially expressed between the two groups. We found 847 DEGs, including 796 upregulated genes and 51 downregulated genes, in the high TMB group compared with the low TMB group. The top 40 most DEGs were visualized by using a heat map (Figure 3A). A volcano map was plotted to exhibit the DEGs (Figure 3B). For GO analysis, we revealed that DEGs were mainly enriched in muscle system process, collagen-containing extracellular matrix and receptor ligand activity processes (Figure 3C). In addition, we conducted KEGG analysis based on DEGs. We found that DEGs mainly belonged to the neuroactive ligand-receptor interaction, cAMP signaling pathway, calcium signaling pathway, and vascular smooth muscle contraction and cell adhesion molecules categories (Figure 3D).

### Construction and Validation of Prognostic Model

**FIGURE 4:**
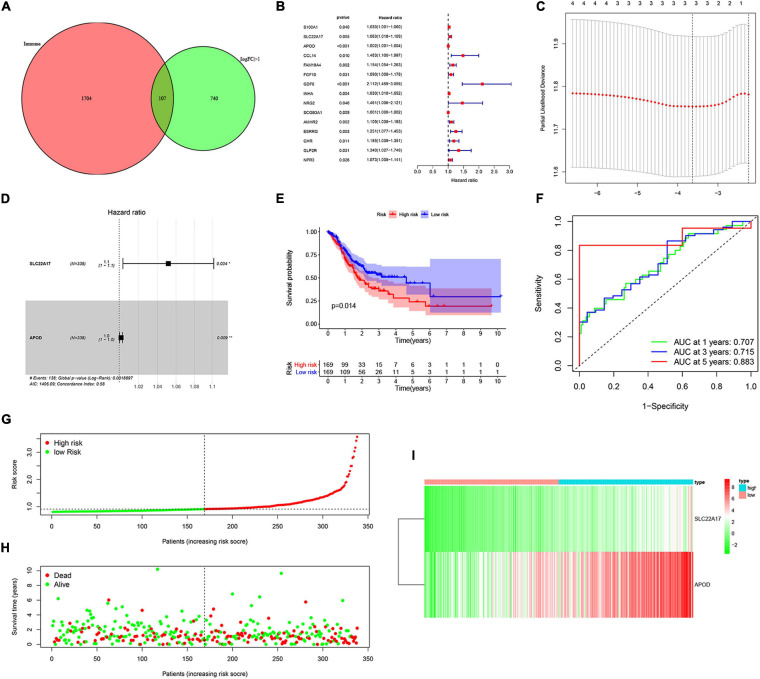
Construction and validation of prognostic model. **(A)** Venn analysis of immune-related differentially expressed genes. **(B)** 15 candidate prognosis-related genes were obtained using univariate analysis. **(C,D)** Two prognosis-related genes were obtained by using LASSO regression and used for the construction of prognostic model. **(E,F)** High-risk group correlated with poor survival outcome, with *p* = 0.014. **(E)** Survival analysis of high-risk and low-risk groups. **(F)** ROC curves of 1, 2-, and 5-year survival prediction, with AUC = 0.707, 0.715, and 0.883, respectively. **(G,H)** The distribution of risk score and gene expression levels among patients in the cancer genome atlas (TCGA) data. **(I)** The expression of two prognostic genes between high-risk and low-risk patients in TCGA training set.

To determine the relationship between TMB and immune infiltration in patients with AGC, we obtained immune-related DEGs by intersecting the 847 DEGs related to TMB with 1881 immune-related genes. A total of 107 immune-related DEGs were identified for further analysis (Figure 4A). Then, we identified 15 genes as candidate prognosis-related genes by using univariate analysis (Figure 4B). The hazard ratio of prognostic genes was shown in Table 1. LASSO regression was subsequently performed on 15 candidate prognosis-related genes, and two genes were retained for constructing the prognostic model (Figures 4C,D). TCGA and GEO data were downloaded to verify the accuracy of the model. We first validated the accuracy of the model in the TCGA dataset. After ranking the patients according to the calculated risk score, patients were divided into a low-risk group and a high-risk group according to the median risk score. Low-risk group patients had better outcomes in terms of survival probability (Figure 4E). A ROC curve was plotted to validate the accuracy of the prognostic model (Figure 4F). Then, patients were ranked based on risk score (Figure 4G). The risk score for each patient was provided in Supplementary Table 4. We found that patients had longer survival times in the low-risk group, and more patients died in the high-risk group (Figure 4H). The expression of the two genes in each group was visualized by a heat map, and gene expression increased in parallel with the risk score (Figure 4I). Then, the GSE84437 data in the GEO database was used as the validation set, and we got similar results (Supplementary Figures 2A–E). This confirmed the reliability of our model.

**TABLE 1:** Univariate COX regression analysis of TMB related prognostic genes in advanced gastric cancer.

Gene symbol	HR	(95%CI)	*p*-Value
*S100A1*	1.0301	(1.0012–1.0597)	0.0404
*SLC22A17*	1.0627	(1.0184–1.1089)	0.0051
*APOD*	1.0022	(1.0009–1.0035)	0.0006
*CCL14*	1.4825	(1.1003–1.9974)	0.0096
*FAM19A4*	1.1536	(1.0536–1.2631)	0.0019
*GDF6*	2.1123	(1.4587–3.0587)	7.52e-05
*INHA*	1.0304	(1.0096–1.0516)	0.0039
*NRG2*	1.4612	(1.0064–2.1214)	0.00160
*SCGB3A1*	1.0012	(1.0003–1.0022)	0.0088
*GHR*	1.1847	(1.0390–1.3509)	0.0113
*GLP2R*	1.3403	(1.0274–1.7486)	0.0307
*NPR3*	1.0725	(1.0083–1.1408)	0.0261
*FGF10*	1.0897	(1.0080–1.1779)	0.0306
*AMHR2*	1.1091	(1.0381–1.1849)	0.0021
*ESRRG*	1.2509	(1.0767–1.4532)	0.0034

*HR, hazard ratio; CI, confidence interval.*

### APOD and SLC22A17 Are Related to Patient Survival, TMB, and Patient Clinical Characteristics

**FIGURE 5:**
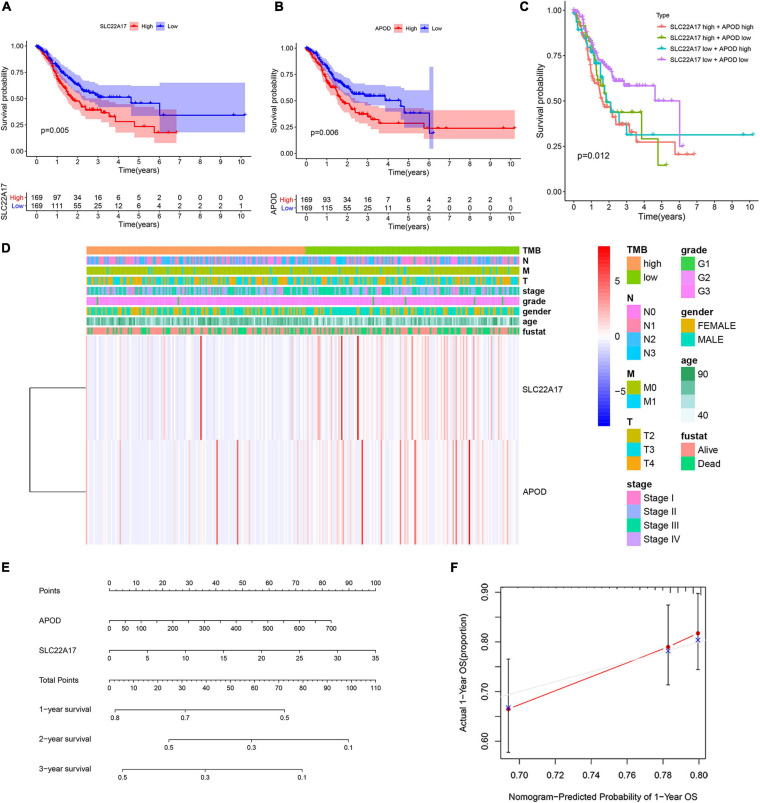
Two genes in prognostic model were associated with patients’ survival and clinical characteristics. **(A,B)** Survival analysis of **(A)** SLC22A17 and **(B)** APOD genes in patients with AGC. **(C)** Survival analysis of AGC patients with different expressions group of SLC22A17 and APOD. **(D)** The expression of SLC22A17 and APOD are associated with patients’ TMB and clinical characteristics. **(E)** The patients’ 1-, 2-, and 3-year survival were predicted by using a nomogram. **(F)** Calibration curves for the survival probability at 1 year.

We obtained two key genes, APOD and SLC22A17, from the prognostic model. To determine whether APOD and SLC22A17 affect the survival probability of patients, we performed K-M survival analysis to explore the survival rates of the two groups. It can be observed that higher expression of ADPO and SLC22A17 correlated with worse prognosis (Figures 5A,B). In addition, we found that patients in both the APOD low group and SLC22A17 low group had the better prognosis. Conversely, if the two genes both are highly expressed at the same time, the patient prognosis is even worse (Figure 5C). The expression of SLC22A17 and APOD in TMB-high and TMB-low group was shown in Supplementary Figure 4. These results indicated that APOD and SLC22A17 can be applied simultaneously for predicting patient prognosis. We further detected the relationship among the expression level of the two genes, TMB and clinical characteristics. The results showed that the expression of the two genes was lower in the high-TMB group (Figure 5D). The relationship between SLC22A17 and APOD gene expression and each clinical feature such as age, gender, grade, stage, and TNM-stage were shown in Supplementary Figure 3. We only found that the expression of SLC22A17 is related to the patient’s age. In addition, a nomogram was further constructed according to the gene expression levels of APOD and SLC22A17 in the TCGA datasets. The patients’ 1-, 2-, and 3-year survival could be predicted by using a nomogram (Figure 5E). At the same time, the calibration curves of the model also confirmed that the predicted 1-year survival rate was relatively consistent with the actual 1-year survival rate (Figure 5F).

### Relation of TMB and Prognostic Model Genes With Immune Cell Infiltration

**FIGURE 6:**
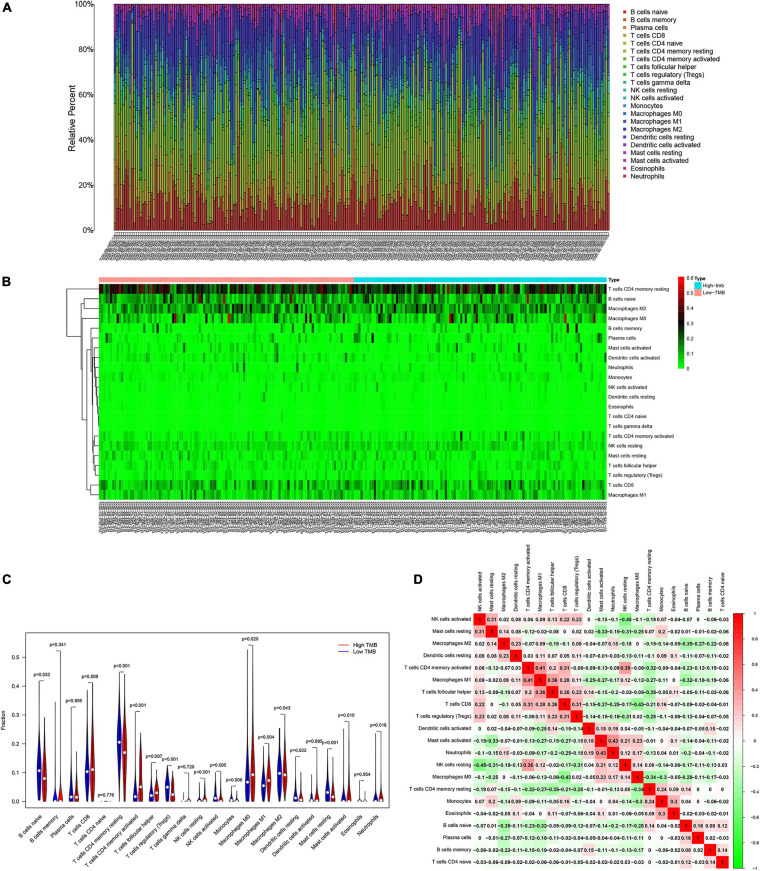
Patients with various degree of TMB have different features of immune cell infiltration. **(A)** Proportion of immune infiltrating cells in gastric cancer samples. **(B)** Heat map and **(C)** bar graph of immune infiltrating cells between high-TMB and low-TMB patients. **(D)** Correlation analysis of 22 kinds of immune cells.

Patients with higher TMB scores have been reported to manifest better response to immunotherapy. However, whether TMB is associated with immune infiltration remains unclear. In order explore the underlying association, we detected the proportions of 22 types of infiltrating immune cells in gastric cancer samples by using the CIBERSORT algorithm. The results are shown in a bar plot map (Figure 6A). Then, we compared the distributions of the 22 types of infiltrating immune cells in the high-TMB and low-TMB groups. The results were visualized in a heat map (Figure 6B). We found that naive B cells, resting memory CD4 T cells, regulatory T cells (Tregs), activated NK cells, monocytes, resting dendritic cells and resting mast cells had higher levels of infiltration in the low-TMB group. In contrast, activated memory T cells, follicular helper T cells, resting NK cells, M0 macrophages, M1 macrophages, activated mast cells, and neutrophils were more abundant in the high-TMB group (Figure 6C). Next, we detected the correlations among 22 types of infiltrating immune cells and visualized them in a matrix based on the Pearson correlation coefficient (Figure 6D).

**FIGURE 7:**
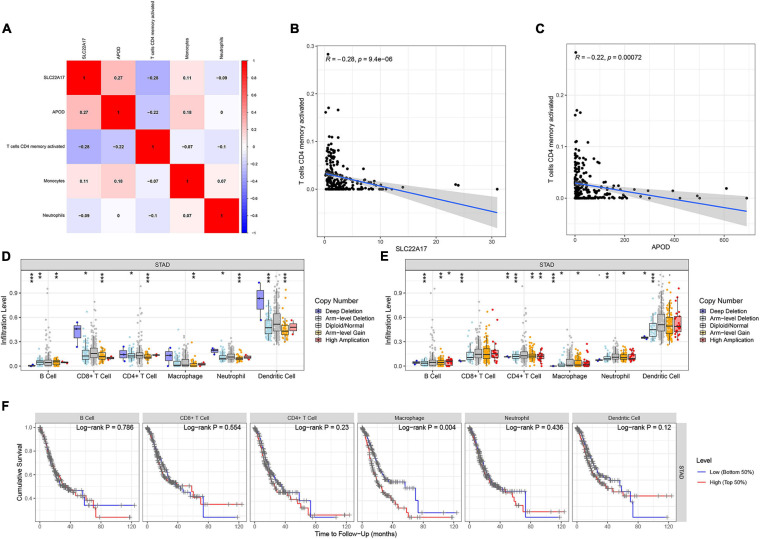
Relation of TMB and prognostic model genes with immune cell infiltration. **(A)** Correlation between prognostic related immune infiltrating cells and prognostic model constructed by SLC22A17 and APOD. **(B,C)** T cells CD4 memory activated was negative associated with the expression of **(B)** SLC22A17 and **(C)** APOD. **(D,E)** Immune infiltration level among gastric cancer patients with diverse degree of copy number variation. **(F)** Survival probability of patients with low and high immune infiltration level of six immune cells. *p < 0.05, **p < 0.01, and ***p < 0.001.

Furthermore, we calculated the correlation between the infiltration of each of the 22 types of immune cells and the expression of APOD and SLC22A17 (Figure 7A). Based on the correlation matrix, we found that APOD (*R* = −0.28, *p* = 9.4E-06) and SLC22A17 (*R* = −0.22, *p* = 0.00072) were negatively associated with T cell CD4 memory activation (Figures 7B,C). The TIMER, containing the abundance of six tumor-infiltrating immune subsets, was further utilized to detect the correlation between copy number variation and the infiltration level of immune cells. We found that the infiltration level was broadly decreased in patients with APOD and SLC22A17 copy number variation compared with the diploid/normal group (Figures 7D,E). To determine whether the infiltration levels of these six immune cells affect patient survival rate, we performed survival analysis to explore the association of immune infiltration with gene expression and survival outcomes. We observed that patients with low levels of macrophage infiltration had better survival outcomes (Figure 7F).

### Evaluation of the Value of TMB-Related Prognostic Model Genes Across Cancers

**FIGURE 8:**
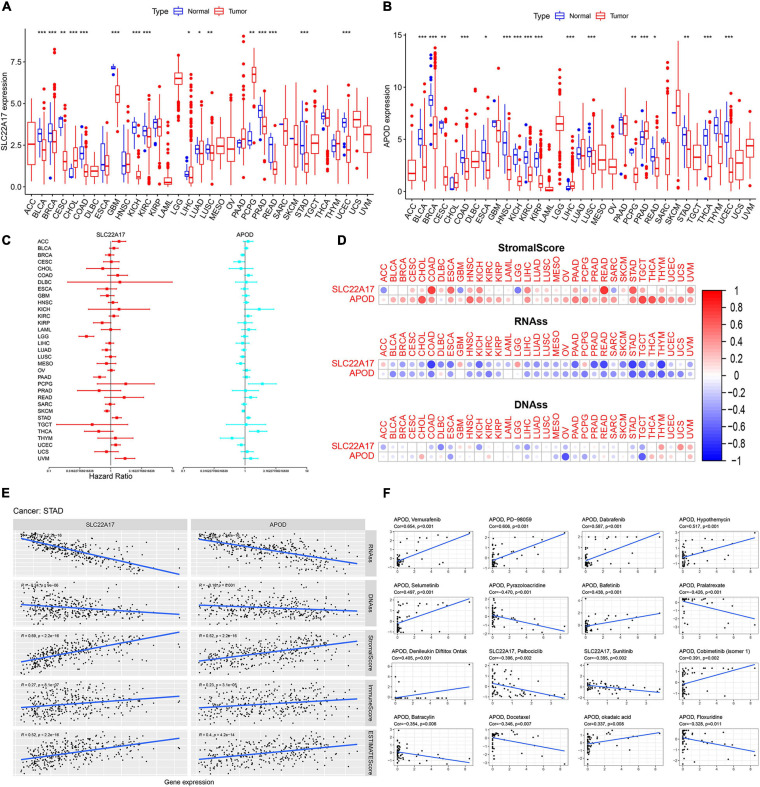
SLC22A17 and APOD are dysregulated in multi-types cancer cells and related to cancer stemness and drug resistance. **(A,B)** Expression of **(A)** SLC22A17 and **(B)** APOD in multi-types cancer cells. **(C)** The prognostic value of SLC22A17 and APOD in the 33 cancers was identified by using univariate cox regression analysis. **(D,E)** SLC22A17 and APOD are associated with cancer stemness in various cancer types, including gastric cancer. **(F)** The correlation between SLC22A17, APOD, and tumor drug resistance. The abscissa and ordinate represent drug sensitivity score and gene expression, respectively. *p < 0.05, **p < 0.01, and ***p < 0.001.

APOD and SLC22A17 are dysregulated and can be used for prognosis in gastric cancer patients. However, whether these two genes exert functions in other cancers is not known. To detect the value of the two genes in other cancers, we downloaded TCGA pancancer data. Then, we analyzed the expression levels of APOD and SLC22A17 in 33 types of cancers. We observed that APOD was dysregulated in 17 types of cancers and that SLC22A17 was dysregulated in 16 types of cancers, with significant *p*-values (Figures 8A,B). Univariate Cox regression analysis was subsequently used to identify the prognostic value in the 33 cancers (Figure 8C). The ESTIMATE algorithm was used to detect the correlation between gene expression and stromal scores, RNAss, and DNAss in 33 different cancer types. Not surprisingly, we found that APOD and SLC22A17 have a wide range of stromal scores in association with 33 different cancer types. In addition, in terms of the correlation between the two genes and cancer stemness, APOD and SLC22A17 had various degrees of association with the RNAss and DNAss in 33 types of cancers (Figure 8D). Interestingly, we observed that the APOD and SLC22A17 genes were negatively correlated with RNAss and DNAss in almost all of the cancer types. In contrast, SLC22A17 and APOD were positively associated with RNAss in patients with ACC, GBM, LGG, PCPG, and DLBC. In addition, SLC22A17 is strongly positively associated with DNAss in GBM, HNSC, THYM, USC, and UVM patients. APOD was strongly positively related to DNAss in CHOL, DLBC, KIRC, READ, SKCM, THCA, THYM, UCEC, and UVM patients.

Pearson correlation was subsequently performed to detect the correlation coefficient between the two genes and RNAss, DNAss, StromalScore, ImmuneScore, and ESTIMATEScore in patients with STAD. The SLC22A17 and APOD genes were negatively associated with RNAss and DNAss, which is consistent with the results of univariate Cox regression analysis. However, SLC22A17 and APOD had positive relationships with the StromalScore, ImmuneScore and ESTIMATEScore (Figure 8E). For the correlation between SLC22A17, APOD, and tumor drug resistance, we next determined the effect of SLC22A17 and APOD on drug sensitivity. Drugs approved by the FDA or drugs in clinical trials were selected for the correlation analysis. Interestingly, APOD exerts a greater role in drug sensitivity analysis. We found that APOD is positively related to sensitivity to vemurafenib, PD-98059, dabrafenib, hypothemycin, selumetinib, bafetinib, denileukin diftitox (Ontak), cobimetinib, and okadaic acid. By contrast, APOD is negatively associated with sensitivity to pyrazoloacridine, pralatrexate, batracylin, docetaxel, and floxuridine. However, SLC22A17 only had a negative relationship with the sensitivity to palbociclib and sunitinib (Figure 8F).

## Discussion

Gastric cancer is a malignant tumor with a high recurrence rate and ranks as the third leading cause of cancer-related death worldwide (Al-Mahrouqi et al., 2011). In recent years, enormous progress has been made in the diagnosis and treatment of gastric cancer. However, the mortality of GC, and especially of AGC, remains high. Therefore, it is of great significance to explore the molecular subtypes of AGC and find effective targeted therapy strategies for specific subtypes.

Gene mutation is closely associated with the initiation and development of cancer (Ikediobi et al., 2006). For example, it has been reported that mutation in BRCA2 is closely related to patient survival, chemotherapy response, and genome instability (Yang et al., 2011). APC mutations are common in colorectal cancers (Nishisho et al., 1991). In addition, mutation of APC is related to the stage of colorectal cancer (Robles et al., 2016). Mutations in cancer-related genes also affect treatment strategies (Hu H. et al., 2018). TMB is a vital biological indicator reflecting the degree of tumor mutation. TMB varies widely among cancer patients. Alexandrov LB reported that TMB could affect the immunotherapy effect of cancer (Alexandrov et al., 2013). Recently, TMB was identified as an immunotherapy biomarker (Chan et al., 2019). With regard to how TMB affects immunotherapy outcomes, Chen DS reported that there are more proteins produced by high-TMB patients, and these proteins can be recognized by the immune system. Immune cells are more easily able to identify and eliminate those tumor cells with high TMB (Chen and Mellman, 2017; Chan et al., 2019). Further research on the association of TMB and immunity will be helpful to identify the critical biomarkers and pathways of AGC.

To explore the association of TMB with AGC, we analyzed somatic mutations in AGC patient samples. A total of 222 (88.45%) patients were identified to have somatic mutations. We ranked the top 30 most common mutations in these patients. The TTN, TP53, and MUC16 genes had the highest mutation frequencies. TTN mutation has been reported to be correlated with prognosis in lung cancer and gastric cancer (Cheng et al., 2019; Yang et al., 2020). MUC16 has also been reported to be associated with prognosis and immunotherapy efficiency in gastric cancer (Yang et al., 2020). TP53 mutation is common and affects treatment strategies in various cancers (Jiao et al., 2018; Kaur et al., 2018; Barbosa et al., 2019; Ahn et al., 2020). The mutant genes are enriched in key pathways involved in cancer progression. The WNT, NOTCH, and RTK-RAS signaling pathways are often dysregulated and can be employed as therapeutic targets in diverse cancers (Nusse and Clevers, 2017; Imperial et al., 2019; Krishna et al., 2019). According to the degree of TMB, we divided patients into a high-TMB group and a low-TMB group. Patients in the high-TMB group had better survival outcomes, which is consistent with the results in other cancers (Devarakonda et al., 2018). Patients aged over 65 have higher TMB. We attributed this to the weak ability of patients aged over 65 to eliminate mutations. The DEGs related to TMB were identified according to the degree of TMB. The results showed that these genes were mainly enriched in neuroactive ligand-receptor interactions, the cAMP signaling pathway and the calcium signaling pathway.

Differentially expressed genes related to TMB were intersected with 1881 immune-related genes. Then, we constructed a prognostic model with two prognostic genes, SLC22A17 and APOD. Based on the prognostic model, TCGA and GEO datasets were used to test the efficiency of the model. As expected, patients in the two low-risk cohorts had better survival outcomes. These results indicated that the prognostic model of differentially expressed TMB-related genes combined with immune-related genes functions well in gastric cancer. In addition, a nomogram was employed to predict the survival rate in gastric cancer. Then, we determined the prognostic function of SLC22A17 and APOD. The relationship between the expression levels of the two genes and patient clinical characteristics was visualized using a heat map. These two genes can be considered prognostic biomarkers in gastric cancer. APOD was reported to be the prognostic factor of gastric. Patients with high expression of APOD might have a shorter OS time. Two authors have also reported that SLC22A17 could be a prognosis biomarker of gastric cancer. Specifically, SLC22A17 was identified as a prognosis gene which may affect immune cell infiltration and iron metabolism in gastric (Hu C. et al., 2018; Wang et al., 2020; Wei et al., 2020). Although these two genes have been reported to be involved in gastric cancer, the specific mechanism of their regulation of gastric cancer is still unclear, which needs further research. In addition, whether these two genes possess prognosis function across different types of cancers remains unclear. Hence, we detected the expression of SLC22A17 and APOD in 33 types of cancers and determined the association of the two genes with cancer stemness-related indicators (Zhang X. et al., 2020). SLC22A17 and APOD were found to be dysregulated in diverse cancers. In almost all cancers, SLC22A17 and APOD have positive relationships with the StromalScore, ImmuneScore and ESTIMATEScore. In contrast, the SLC22A17 and APOD genes were negatively associated with RNAss and DNAss in most cancers. Regarding drug resistance, we observed that APOD exerted a greater role in drug sensitivity. APOD has a strong positive relationship with resistance to many drugs. All these results indicated that these two genes have the same expression pattern and exhibit a similar correlation with StromalScore, RNAss, and DNAss in nearly all cancers. However, the predictive performance of these genes for other specific cancers requires more research.

Tumor mutation burden affects the degree of immune infiltration and efficacy of immune therapy in several cancers (Wu et al., 2019; Kang et al., 2020; Zhang L. et al., 2020). To explore the underlying association in gastric cancer, we analyzed the distribution of 22 infiltrating immune cells in tumor samples. The results showed that the proportions of infiltrating immune cells varied between the high-TMB group and the low-TMB group. Some kinds of infiltrating immune cells increased in tumor samples with high TMB. However, numerous infiltrating immune cells were decreased in tumor samples with low TMB. More research is needed to determine whether the infiltration of each type of immune cell is caused by TMB. To further clarify the association of TMB and immune infiltration in AGC, we analyzed the immune infiltration level in samples with diverse TMBs and found that the infiltration level was broadly decreased in patients with higher copy number variation compared with the diploid/normal group, which is consistent with other studies (Hu H. et al., 2018; Chan et al., 2019). Interestingly, we observed that patients with low infiltration had better survival outcomes. We speculate that this may be related to the poor prognosis of patients with AGC; the stage of patients diagnosed with AGC and the available therapeutic strategies may also account for this difference. More experiments are needed to clarify the association between TMB and immune infiltration.

## Conclusion

Our results indicate that immune-related genes generated from TMB-related differential expression analysis are involved in the progression of AGC. A prognostic model constructed with SLC22A17 and APOD might have vital roles across multiple types of cancers. Detection of TMB combined with immune infiltrating cells in AGC patients could be an effective method in guiding cancer therapy strategies, especially immunotherapy.

## Data Availability Statement

The datasets presented in this study can be found in online repositories. The names of the repository/repositories and accession number(s) can be found in the article/Supplementary Material.

## Author Contributions

XG, YW, and XL designed the study. XG, XL, CQ, and HZ collected and analyzed the data. XL, XG, and AC wrote and revised the manuscript. ZW was responsible for supervising the study. All authors read and gave final approval of the manuscript.

## Conflict of Interest

The authors declare that the research was conducted in the absence of any commercial or financial relationships that could be construed as a potential conflict of interest.

## Footnotes

1 https://portal.gdc.cancer.gov/

2 https://www.immport.org/

3 https://cistrome.shinyapps.io/timer/

4 https://xenabrowser.net/datapages/

5 http://bioinformatics.mdanderson.org/estimate/

## References

[B1] Ahn I. Tian X. Wiestner A. (2020). TP53Ibrutinib for chronic lymphocytic leukemia with alterations. *N. Engl. J. Med.* 383 498–500. 10.1056/NEJMc2005943 32726539 PMC7456330

[B2] Ajani J. Lee J. Sano T. Janjigian Y. Fan D. Song S. (2017). Gastric adenocarcinoma. *Nat. Rev. Dis. Prim.* 3:17036. 10.1038/nrdp.2017.36 28569272

[B3] Alexandrov L. Nik-Zainal S. Wedge D. Aparicio S. Behjati S. Biankin A. (2013). Signatures of mutational processes in human cancer. *Nature* 500 415–421. 10.1038/nature12477 23945592 PMC3776390

[B4] Al-Mahrouqi H. Parkin L. Sharples K. (2011). Incidence of stomach cancer in oman and the other gulf cooperation council countries. *Oman Med. J.* 26 258–262. 10.5001/omj.2011.62 22043430 PMC3191710

[B5] Barbosa K. Li S. Adams P. Deshpande A. (2019). The role of TP53 in acute myeloid leukemia: challenges and opportunities. *Genes Chromos. Cancer* 58 875–888. 10.1002/gcc.22796 31393631 PMC12042961

[B6] Bi F. Chen Y. Yang Q. (2020). Significance of tumor mutation burden combined with immune infiltrates in the progression and prognosis of ovarian cancer. *Cancer Cell Int.* 20:373. 10.1186/s12935-020-01472-9 32774167 PMC7405355

[B7] Cascinu S. (2020). Lenvatinib and pembrolizumab in advanced gastric cancer. *Lancet Oncol.* 21 1004–1005. 10.1016/s1470-2045(20)30336-3 32589865

[B8] Chan T. A. Yarchoan M. Jaffee E. Swanton C. Quezada S. A. Stenzinger A. (2019). Development of tumor mutation burden as an immunotherapy biomarker: utility for the oncology clinic. *Ann. Oncol.* 30 44–56. 10.1093/annonc/mdy495 30395155 PMC6336005

[B9] Chen L.-L. (2016). The biogenesis and emerging roles of circular RNAs. *Nat. Rev. Mol. Cell Biol.* 17 205–211. 10.1038/nrm.2015.32 26908011

[B10] Chen D. Mellman I. (2017). Elements of cancer immunity and the cancer-immune set point. *Nature* 541 321–330. 10.1038/nature21349 28102259

[B11] Cheng X. Yin H. Fu J. Chen C. An J. Guan J. (2019). Aggregate analysis based on TCGA: TTN missense mutation correlates with favorable prognosis in lung squamous cell carcinoma. *J. Cancer Res. Clin. Oncol.* 145 1027–1035. 10.1007/s00432-019-02861-y 30810839 PMC11810161

[B12] Devarakonda S. Rotolo F. Tsao M. Lanc I. Brambilla E. Masood A. (2018). Tumor mutation burden as a biomarker in resected non-small-cell lung cancer. *J. Clin. Oncol. Off. J. Am. Soc. Clin. Oncol.* 36 2995–3006. 10.1200/jco.2018.78.1963 30106638 PMC6804865

[B13] Guo X. Wang Y. Zhang H. Qin C. Cheng A. Liu J. (2020). Identification of the prognostic value of immune-related genes in esophageal cancer. *Front. Genet.* 11:989. 10.3389/fgene.2020.00989 32973887 PMC7472890

[B14] Hu C. Zhou Y. Liu C. Kang Y. (2018). A novel scoring system for gastric cancer risk assessment based on the expression of three CLIP4 DNA methylation-associated genes. *Int. J. Oncol.* 53 633–643. 10.3892/ijo.2018.4433 29901187 PMC6017186

[B15] Hu H. Mu Q. Bao Z. Chen Y. Liu Y. Chen J. (2018). Mutational landscape of secondary glioblastoma guides MET-targeted trial in brain tumor. *Cell* 175 1665–1678.e1618. 10.1016/j.cell.2018.09.038 30343896

[B16] Ikediobi O. Davies H. Bignell G. Edkins S. Stevens C. O’Meara S. (2006). Mutation analysis of 24 known cancer genes in the NCI-60 cell line set. *Mol. Cancer Therap.* 5 2606–2612. 10.1158/1535-7163.mct-06-0433 17088437 PMC2705832

[B17] Imperial R. Toor O. Hussain A. Subramanian J. Masood A. (2019). Comprehensive pancancer genomic analysis reveals (RTK)-RAS-RAF-MEK as a key dysregulated pathway in cancer: Its clinical implications. *Semin. Cancer Biol.* 54 14–28. 10.1016/j.semcancer.2017.11.016 29175106

[B18] Jiang T. Shi J. Dong Z. Hou L. Zhao C. Li X. (2019). Genomic landscape and its correlations with tumor mutational burden, PD-L1 expression, and immune cells infiltration in Chinese lung squamous cell carcinoma. *J. Hematol. Oncol.* 12:75. 10.1186/s13045-019-0762-1 31299995 PMC6625041

[B19] Jiao X. Qin B. You P. Cai J. Zang Y. (2018). The prognostic value of TP53 and its correlation with EGFR mutation in advanced non-small cell lung cancer, an analysis based on cBioPortal data base. *Lung cancer (Amster. Nether.)* 123 70–75. 10.1016/j.lungcan.2018.07.003 30089598

[B20] Kang K. Xie F. Mao J. Bai Y. Wang X. (2020). Significance of tumor mutation burden in immune infiltration and prognosis in cutaneous melanoma. *Front. Oncol.* 10:573141. 10.3389/fonc.2020.573141 33072607 PMC7531222

[B21] Kaur R. Vasudeva K. Kumar R. Munshi A. (2018). Role of p53 gene in breast cancer: focus on mutation spectrum and therapeutic strategies. *Curr. Pharmaceut. Design* 24 3566–3575. 10.2174/1381612824666180926095709 30255744

[B22] Kawazoe A. Shitara K. Boku N. Yoshikawa T. Terashima M. (2020). Current status of immunotherapy for advanced gastric cancer. *Jpn. J. Clin. Oncol.* 51 20–27. 10.1093/jjco/hyaa202 33241322

[B23] Kim H. J. Oh S. C. (2018). Novel systemic therapies for advanced gastric cancer. *J. Gastric. Cancer* 18 1–19. 10.5230/jgc.2018.18.e3 29629216 PMC5881006

[B24] Krishna B. Jana S. Singhal J. Horne D. Awasthi S. Salgia R. (2019). Notch signaling in breast cancer: From pathway analysis to therapy. *Cancer Lett.* 461 123–131. 10.1016/j.canlet.2019.07.012 31326555 PMC9003668

[B25] Le D. Uram J. Wang H. Bartlett B. Kemberling H. Eyring A. (2015). PD-1 blockade in tumors with mismatch-repair deficiency. *N. Engl. J. Med.* 372 2509–2520. 10.1056/NEJMoa1500596 26028255 PMC4481136

[B26] Mayakonda A. Lin D. Assenov Y. Plass C. Koeffler H. (2018). Maftools: efficient and comprehensive analysis of somatic variants in cancer. *Genome Res.* 28 1747–1756. 10.1101/gr.239244.118 30341162 PMC6211645

[B27] Morrison C. Pabla S. Conroy J. Nesline M. Glenn S. Dressman D. (2018). Predicting response to checkpoint inhibitors in melanoma beyond PD-L1 and mutational burden. *J. Immunother. Cancer* 6:32. 10.1186/s40425-018-0344-8 29743104 PMC5944039

[B28] Nishisho I. Nakamura Y. Miyoshi Y. Miki Y. Ando H. Horii A. (1991). Mutations of chromosome 5q21 genes in FAP and colorectal cancer patients. *Science (New York N. Y.)* 253 665–669. 10.1126/science.1651563 1651563

[B29] Nusse R. Clevers H. (2017). Wnt/β-catenin signaling, disease, and emerging therapeutic modalities. *Cell* 169 985–999. 10.1016/j.cell.2017.05.016 28575679

[B30] Robles A. Traverso G. Zhang M. Roberts N. Khan M. Joseph C. (2016). Whole-exome sequencing analyses of inflammatory bowel disease-associated colorectal cancers. *Gastroenterology* 150 931–943. 10.1053/j.gastro.2015.12.036 26764183 PMC5270616

[B31] Song Z. Wu Y. Yang J. Yang D. Fang X. (2017). Progress in the treatment of advanced gastric cancer. *Tumour. Biol.* 39:1010428317714626. 10.1177/1010428317714626 28671042

[B32] Tian Y. Xu J. Chu Q. Duan J. Zhang J. Bai H. (2020). A novel tumor mutational burden estimation model as a predictive and prognostic biomarker in NSCLC patients. *BMC Med.* 18:232. 10.1186/s12916-020-01694-8 32843031 PMC7448445

[B33] Wang M. Li Z. Peng Y. Fang J. Fang T. Wu J. (2020). Identification of immune cells and mRNA associated with prognosis of gastric cancer. *BMC Cancer* 20:206. 10.1186/s12885-020-6702-1 32164594 PMC7068972

[B34] Wei J. Gao X. Qin Y. Liu T. Kang Y. (2020). An iron metabolism-related SLC22A17 for the prognostic value of gastric cancer. *Onco. Targets Ther.* 13 12763–12775. 10.2147/ott.S287811 33363382 PMC7751842

[B35] Wu Y. Xu J. Du C. Wu Y. Xia D. Lv W. (2019). The predictive value of tumor mutation burden on efficacy of immune checkpoint inhibitors in cancers: a systematic review and meta-analysis. *Front. Oncol.* 9:1161. 10.3389/fonc.2019.01161 31750249 PMC6848266

[B36] Yang D. Khan S. Sun Y. Hess K. Shmulevich I. Sood A. (2011). Association of BRCA1 and BRCA2 mutations with survival, chemotherapy sensitivity, and gene mutator phenotype in patients with ovarian cancer. *JAMA* 306 1557–1565. 10.1001/jama.2011.1456 21990299 PMC4159096

[B37] Yang Y. Zhang J. Chen Y. Xu R. Zhao Q. Guo W. (2020). MUC4, MUC16, and TTN genes mutation correlated with prognosis, and predicted tumor mutation burden and immunotherapy efficacy in gastric cancer and pan-cancer. *Clin. Transl. Med.* 10:e155. 10.1002/ctm2.155 32898332 PMC7443139

[B38] Yarchoan M. Hopkins A. Jaffee E. (2017). Tumor mutational burden and response rate to PD-1 inhibition. *N. Engl. J. Med.* 377 2500–2501. 10.1056/NEJMc1713444 29262275 PMC6549688

[B39] Yu G. Wang L. Han Y. He Q. (2012). clusterProfiler: an R package for comparing biological themes among gene clusters. *Omics J. Integrat. Biol.* 16 284–287. 10.1089/omi.2011.0118 22455463 PMC3339379

[B40] Zhang L. Li B. Peng Y. Wu F. Li Q. Lin Z. (2020). The prognostic value of TMB and the relationship between TMB and immune infiltration in head and neck squamous cell carcinoma: a gene expression-based study. *Oral oncology* 110:104943. 10.1016/j.oraloncology.2020.104943 32919362

[B41] Zhang X. Klamer B. Li J. Fernandez S. Li L. (2020). A pan-cancer study of class-3 semaphorins as therapeutic targets in cancer. *BMC Med. Genom.* 13:45. 10.1186/s12920-020-0682-5 32241267 PMC7118829

