# Supplementary material for: Significance of Tumor Mutation Burden Combined With Immune Infiltrates in the Progression and Prognosis of Advanced Gastric Cancer
Source: Front Genet. 2021 Jul 9;12:642608. doi: 10.3389/fgene.2021.642608 (PMC8299211; doi:10.3389/fgene.2021.642608)

Supplementary Figure S1. Analyses of somatic mutation profiles in advanced gastric cancer. (A-C) According to different classification categories, missense mutation, SNP, and C > T mutation accounted for the overwhelming majority. The abscissa is the number of bases. (D) The total mutation number in each sample. (E) Box plots of each variant classification in each sample. (F) Top 10 mutated genes in melanoma. SNP, single nucleotide polymorphism; SNV, single nucleotide variants. (G) Fraction of key genes in the pathway affected by mutations. (H) Proportion of samples with mutations in key signaling pathways. (I-K) The waterfall diagram shows the profile of mutations in the (I) RTK-RAS, (J) WNT and (K) NOTCH pathway.

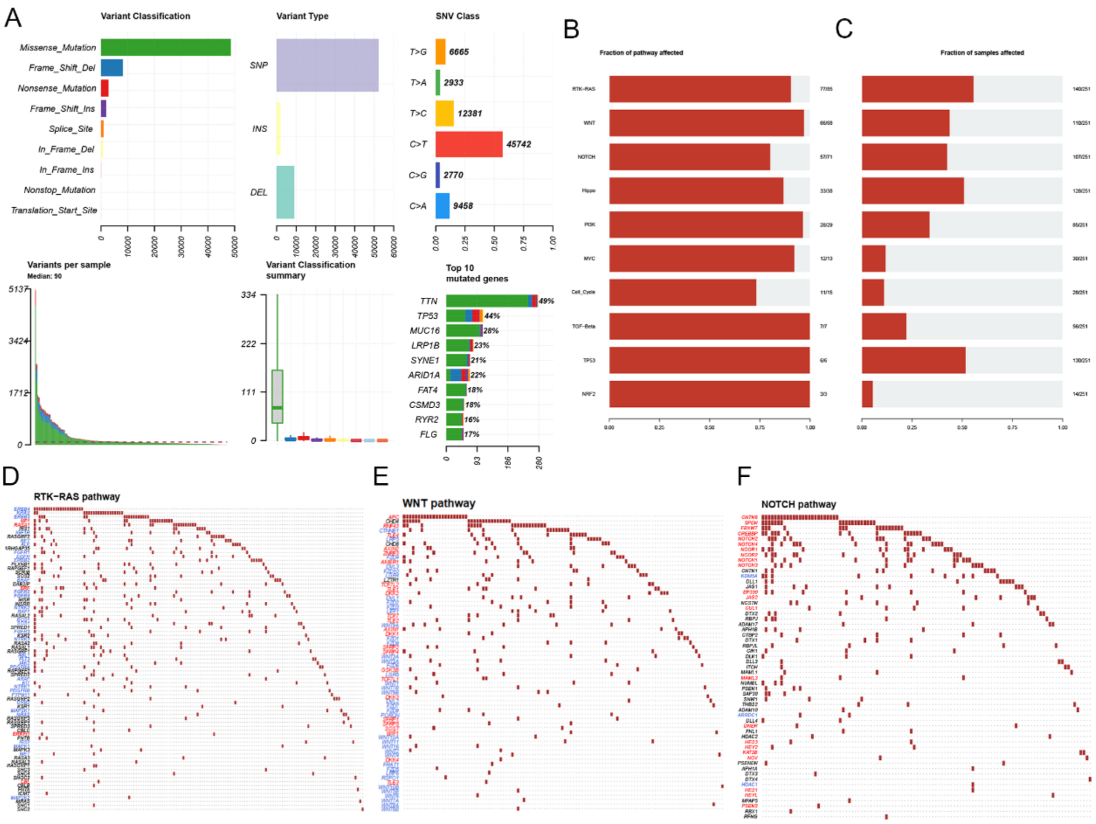

Supplementary Figure S2. The validation data set in GEO database. (A) The high- and low-risk groups of survival analysis are in the GEO validation set. (B) ROC curves of 1, 2- and 5-year survival prediction, with AUC = 0.525, 0.563 and 0.587, respectively. (C and D) The distribution of risk score and gene expression levels among patients in TCGA data. (E) The expression of two prognostic genes between high-TMB and low-TMB patients in TCGA training set.

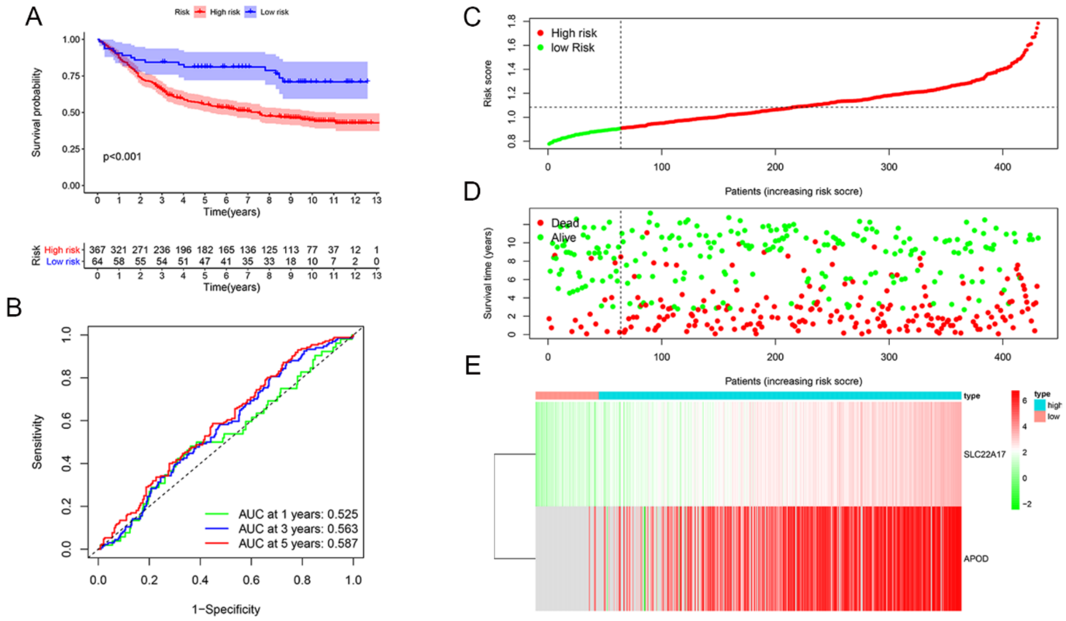

Supplementary Figure S3. The gene expression in the TMB-high and low groups. (A and B) The expression of (A) SLC22A17 and (B) APOD in the TMB-high and low groups.

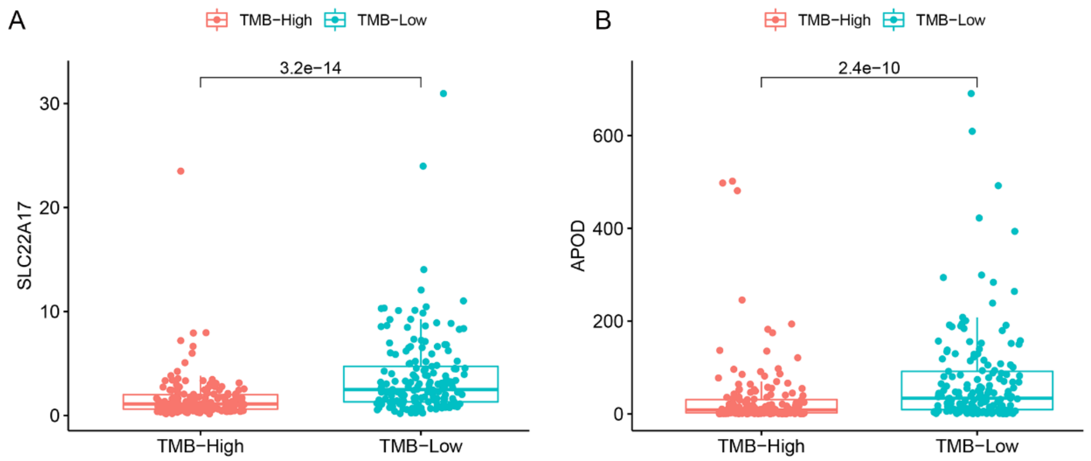

Supplementary Figure S4. Analysis of the correlation between gene expression and clinicopathological characteristics. (A-G) Correlation analysis of SLC22A17 with (A) age, (B) gender, (C) grade, (D) stage, (E) T-stage, (F)N-stage and (G) M-stage. (H-N) Correlation analysis of APOD with (H) age, (I) gender, (J) grade, (K) stage, (L) T-stage, (M) N-stage and (N) M-stage.

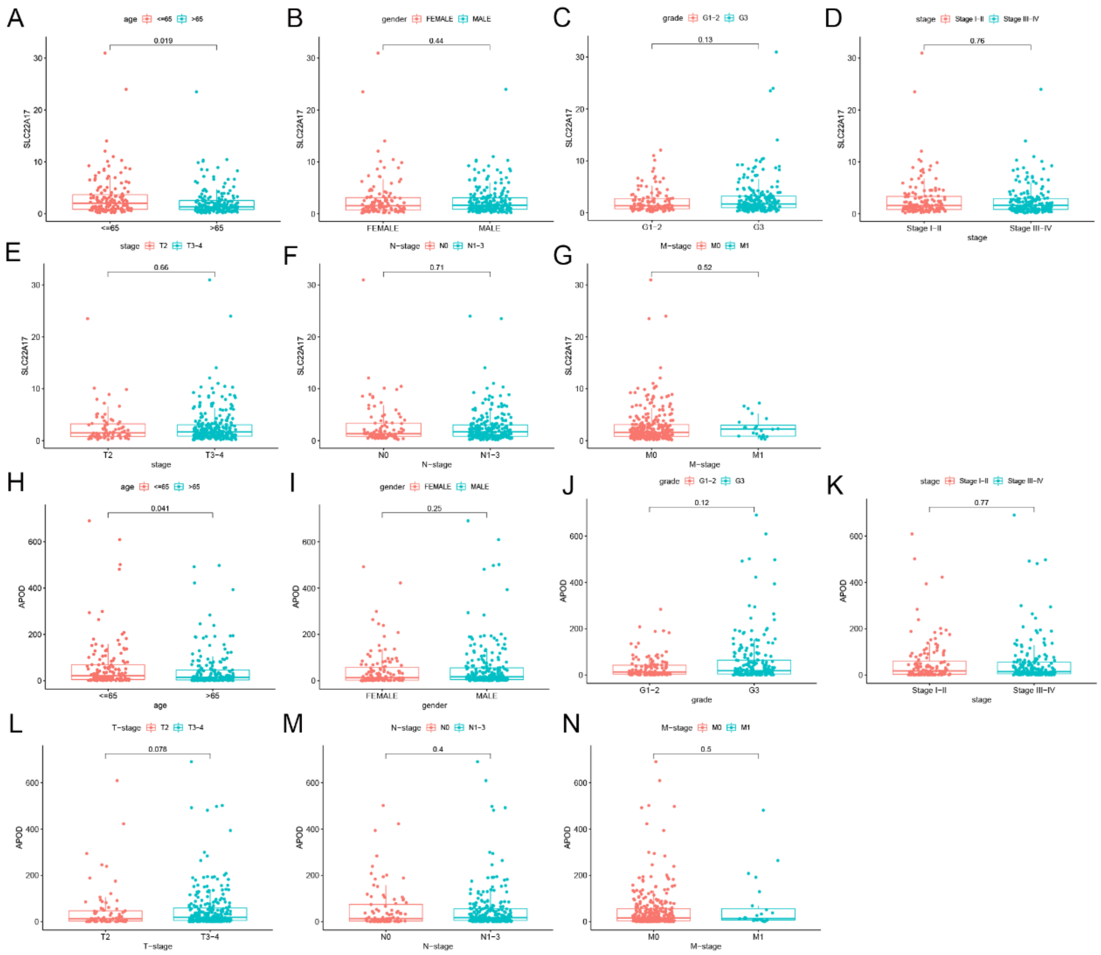

Supplement: Supplementary file 1 [file Data_Sheet_1.PDF]
